# Editorial: Continuous Biomanufacturing in Microbial Systems

**DOI:** 10.3389/fbioe.2021.665940

**Published:** 2021-06-07

**Authors:** Christoph Herwig, Christoph Slouka, Peter Neubauer, Frank Delvigne

**Affiliations:** ^1^Research Division Biochemical Engineering, Institute of Chemical, Environmental and Bioscience Engineering, Vienna University of Technology, Vienna, Austria; ^2^Competence Center CHASE GmbH, Linz, Austria; ^3^Chair of Bioprocess Engineering, Department of Biotechnology, Technische Universität Berlin, Berlin, Germany; ^4^TERRA Teaching and Research Centre, Microbial Processes and Interactions (MiPI), Gembloux Agro-Bio Tech – Université de Liège, Gembloux, Belgium

**Keywords:** continuous bioprocessing, microbial process, cellular mechanism, metabolic load, product location, product recovery, USP, DSP

Continuous processing is without doubt the most effective and economically viable processing mode, because it optimally uses the installed assets. While most chemical industrial processes are designed and executed already in continuous operation, the biotechnology industry still relies on batchwise production, due to multiple unknown variables of complex biological systems.

Recent reports have demonstrated, specifically in the area of cell culture processes, that continuous biomanufacturing (CBM) has significant economic advantages. Continuous processes significantly influence the ecological and economic footprint, as facilities become smaller and versatile with the application of modular design approaches. Continuous cultivation approaches have the need to tightly control process variability along prolonged processing times, calling for accurate process analytics and robust control strategies.

However, establishing integrated processes for production with dedicated microbial systems is far more complicated, although of course continuous processes are standard, e.g., in the areas of biogas production, wastewater treatment and bioleaching. The complication is due to the fact that, especially in the area of the production of defined recombinant proteins, the protein often accumulates in the cell, which represents a natural physical barrier contradicting continuous operational strategies. In addition, common induction systems have a highly time dependent effect and may lead to high temporary metabolic load. Both effects lead to limitations in prolonged recombinant protein production, seen in an irreversible drop in protein productivity. Hence, the lack of basic knowledge about the regulatory mechanisms leading to population evolution during prolonged recombinant protein production prevents the industrial application of continuous biomanufacturing. But also in other microbial processes, with both engineered and non-engineered single-species microbial cell factories, continuous operation has so far rarely been realized.

So what are the current challenges and scientific enablers for advancing continuous biomanufacturing with microbial cells? Several challenges needs to be answered by scientific knowledge (Figure 1, outer circle):

- How to control metabolic load for sustained recombinant product production, for example by the assessment of population stability, analyzing phenotypic, and genotypic instabilities
- How to change the product location to circumvent the internal physical limit of the cell?
- How to achieve prolonged time invariant processing providing stable productivity and product quality to the downstream process
- How to efficiently integrate upstream and down stream processing?

**Figure 1:**
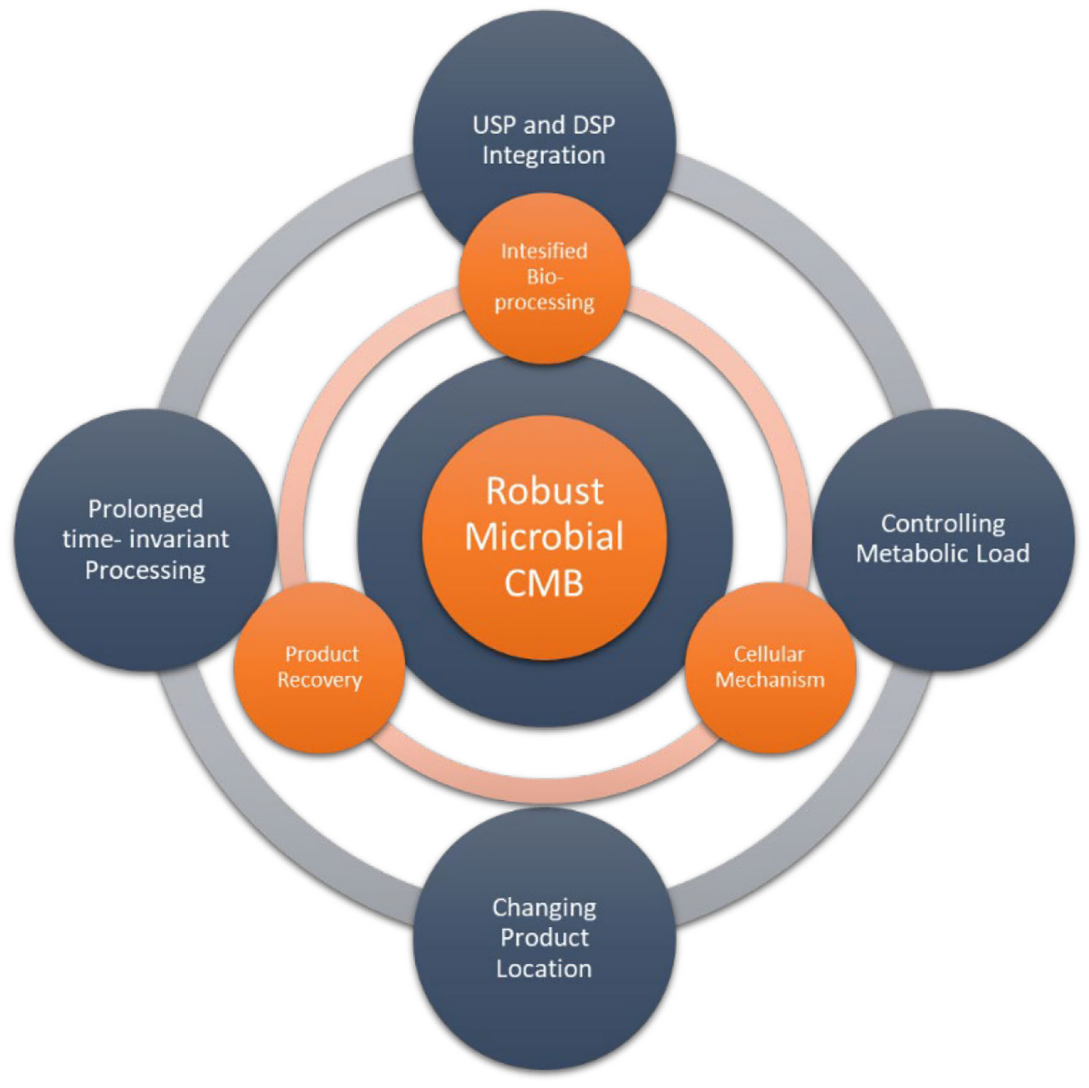
Challenges (outer circle) and in the current topic addressed scientific advancements (inner circle) for enabling robust microbial continuous biomanufacturing (CBM).

The goal of this electronic article collection is to give an overview on recent scientific advances addressing above points.

The selected contributions in this Research Topic show significant scientific advancements to microbial CBM and can be classified in the following three aspects (Figure 1 inner circle):

1. Cellular mechanisms
2. Intensified bioprocessing
3. Product recovery.

Those contributions address well the challenges mentioned above.

## Cellular Mechanisms

It is important to understand the population dynamics and its mechanisms of the cell being cultivated in the bioreactor. The perspective article “Single-Cell Technologies to Understand the Mechanisms of Cellular Adaptation in Chemostats” by Risager Wright et al. addresses the importance that population heterogeneity should be taken into account for CBM design and analyzed the suitability of current analytical devices. The contribution “The Lazarus *Escherichia coli* Effect: Recovery of Productivity on Glycerol/Lactose Mixed Feed in Continuous Biomanufacturing” by Kittler et al. addresses a sudden phenomenon of productivity drop and suggests that the mixed feed of Glycerol and Lactose, but also Galactose as a light inducer, are responsible for population dynamics. Graf et al., with their contribution “Revisiting the Growth Modulon of *Corynebacterium glutamicum* Under Glucose Limited Chemostat Conditions,” analyze the control mechanisms on transcriptomic and metabolic level, with the conclusion, that glycolysis, pentose-phosphate pathway and citric acid cycle are predominately metabolically controlled under glucose-limiting chemostat conditions and that transcriptional regulation takes control over glycolysis once glucose-rich growth conditions are installed.

## Processing Alternatives

How to achieve prolonged sustained productivities in the bioreactor? Challenges such as population heterogeneity, loss of the catalyst need to be overcome. In cell culture processes, perfusion techniques are the method of choice currently.

The research article “Repetitive Fed-Batch: A Promising Process Mode for Biomanufacturing With *E. coli*” by Kopp et al. found that a repetitive fed-batch for *E. coli* leads to a higher space-time yield compared to a single-cycle fed-batch and can potentially outperform continuous biomanufacturing by classical chemostats. Raveschot et al., with their contribution “Integrated Continuous Bioprocess Development for ACE-Inhibitory Peptide Production by *Lactobacillus helveticus* Strains in Membrane Bioreactor” developed an integrated continuous process for peptide production with a membrane bioreactor, which led to a 3 fold increased peptide productivity compared to batch production. In the contribution “The Impact of Glass Material on Growth and Biocatalytic Performance of Mixed-Species Biofilms in Capillary Reactors for Continuous Cyclohexanol Production,” Heuschkel et al. growth and catalytic performance of mixed-species biofilms for cyclohexanol was investigated. Four phases of the biofilm cultivation could be distinguished and the cyclohexanol production rate could be significantly improved.

## Product Recovery

How to deliver continuous product to the downstream? In addition to the production mode, the product must be efficiently be separated from the cells in order to integrate smoothly to the subsequent down stream process.

Pekarsky and Spadiut with their review “Intrinsically Magnetic Cells: A Review on Their Natural Occurrence and Synthetic Generation” investigate the state of the art of Natural intrinsically magnetic cells which can be used to separate the cells during CBM. The research article “Selective release of recombinant periplasmic protein from E. coli using continuous pulsed electric field treatment” by Schottroff et al. focusses on novel process methods to circumvent product accumulation in E. coli and triggering product secretion to the extracellular medium by applying pulsed electric fields. Thakur et al., with their contribution “Automation of Dead End Filtration: An Enabler for Continuous Processing of Biotherapeutics” demonstrated that Dead end filtration, which is conventionally done in batch mode, can be automated and can therefore server as a potential tool for continuous biomanufacturing.

We believe, this topic issue made a significant contribution to advance microbial CBM to become reality. However, we are aware that many more aspects need to be addressed to complete the picture, such as PAT, modeling, and control aspects!

## Author Contributions

CH designed and wrote the manuscript. FD, PN, and CS revived and revised the manuscript. All authors contributed to the article and approved the submitted version.

## Conflict of Interest

The authors declare that the research was conducted in the absence of any commercial or financial relationships that could be construed as a potential conflict of interest.

